# Dibromido[1,1′-dibenzyl-2,2′-(sulfane­diyl­dimethyl­ene)di-1*H*-benzimidazole]­cadmium(II) dimethyl­formamide solvate

**DOI:** 10.1107/S1600536810028448

**Published:** 2010-07-21

**Authors:** Kaitong Wang, Jingkun Yuan, Guisheng Chen, Qian Chen, Huilu Wu

**Affiliations:** a School of Chemical and Biological Engineering, Lanzhou Jiaotong University, Lanzhou 730070, People’s Republic of China

## Abstract

In the title compound, [CdBr_2_(C_30_H_26_N_4_S)]·C_3_H_7_NO, both the complex and solvent mol­ecule lie on a crystallographic mirror plane. The Cd^II^ ion is coordinated in a disorted square-pyramidal CdBr_2_N_2_S environment with one of the Br atoms in the apical site. In the crystal structure, the benzimidazole ring systems are involved in weak inter­molecular π–π stacking inter­actions [centroid–centroid distances = 3.606 (2) and 3.753 (2) Å]. Further stabilization is provided by weak inter­molecular C—H⋯O hydrogen bonds. The methyl H atoms of the dimethyl­formamide solvent mol­ecule are disordered about a mirror plane.

## Related literature

For background to the synthesis and for related structures of 1,3-bis­(benzimidazol-2-yl)-2-thia­propane and its derivatives, see: Dagdigian *et al.* (1979 ▶); Agh-Atabay *et al.*(2004 ▶); Wu *et al.* (2009 ▶).
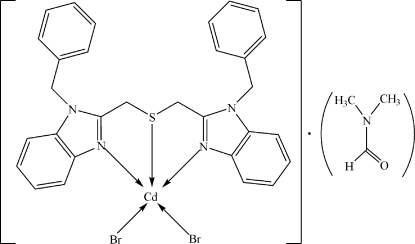


## Experimental

### 

#### Crystal data

[CdBr_2_(C_30_H_26_N_4_S)]·C_3_H_7_NO
*M* *_r_* = 819.92
Monoclinic, 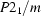

*a* = 9.7437 (8) Å
*b* = 16.7792 (14) Å
*c* = 10.5931 (9) Å
β = 110.029 (1)°
*V* = 1627.1 (2) Å^3^
*Z* = 2
Mo *K*α radiation
μ = 3.23 mm^−1^
*T* = 296 K
0.36 × 0.32 × 0.28 mm

#### Data collection

Bruker APEXII area-detector diffractometer
Absorption correction: multi-scan (*SADABS*; Bruker, 2006 ▶) *T*_min_ = 0.390, *T*_max_ = 0.465
9062 measured reflections
3305 independent reflections
2742 reflections with *I* > 2σ(*I*)
*R*_int_ = 0.027

#### Refinement

*R*[*F* ^2^ > 2σ(*F* ^2^)] = 0.029
*wR*(*F* ^2^) = 0.077
*S* = 1.05
3305 reflections
211 parameters
H-atom parameters constrained
Δρ_max_ = 0.78 e Å^−3^
Δρ_min_ = −0.64 e Å^−3^

### 

Data collection: *APEX2* (Bruker, 2006 ▶); cell refinement: *SAINT* (Bruker, 2006 ▶); data reduction: *SAINT*; program(s) used to solve structure: *SHELXS97* (Sheldrick, 2008 ▶); program(s) used to refine structure: *SHELXL97* (Sheldrick, 2008 ▶); molecular graphics: *SHELXTL* (Sheldrick, 2008 ▶); software used to prepare material for publication: *SHELXTL*.

## Supplementary Material

Crystal structure: contains datablocks global, I. DOI: 10.1107/S1600536810028448/lh5084sup1.cif

Structure factors: contains datablocks I. DOI: 10.1107/S1600536810028448/lh5084Isup2.hkl

Additional supplementary materials:  crystallographic information; 3D view; checkCIF report

## Figures and Tables

**Table 1:** Hydrogen-bond geometry (Å, °)

*D*—H⋯*A*	*D*—H	H⋯*A*	*D*⋯*A*	*D*—H⋯*A*
C1—H1*A*⋯O1^i^	0.97	2.38	3.004 (5)	122

Symmetry code: (i) 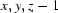.

**Fig. 1.**
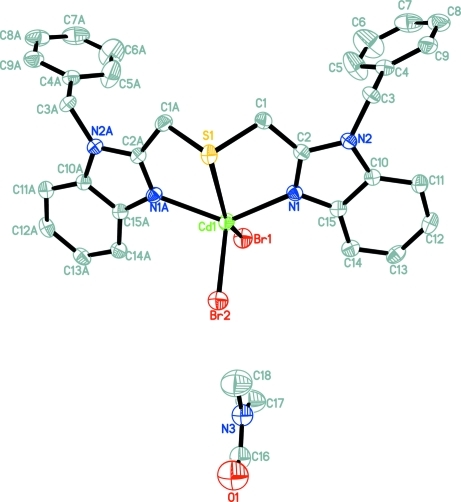
The asymmetric unit of the title compound. Displacement ellipsoids are drawn at the 30% probability level. H atoms have been omitted for clarity [symmetry code (A): x, -y+1/2, z].

## supplementary crystallographic information

### Comment

The asymmetric unit of the title complex is shown in Fig. 1. The Cd^II^ ion is coordinated by one tridentate 1,3-bis(1-benzylbenzimidazol-2-yl)-2-thiapropane ligand and two bromide ions in a distorted square-pyramidal geometry. In the crystal structure, the benzimidazole ring systems are involved in weak intermolecular π–π stacking interactions [centroid–centroid distances = 3.606 (2) and 3.753 (2) Å].

### Experimental

To a stirred solution of 1,3-bis(1-benzylbenzimidazol-2-yl)-2-thiapropane (0.237 g, 0.50 mmol) in hot MeOH (10 ml) was added Cd(C_6_H_2_N_3_O_7_)_2_ (0.154 g, 0.25 mmol) and KBr(0.059 g, 0.50 mmol) in MeOH (5 ml). A yellow crystalline product formed rapidly. The precipitate was filtered off, washed with MeOH and absolute Et_2_O, and dried *in vacuo*. The dried precipitate was dissolved in DMF resulting in a yellow solution. The deep yellow crystals suitable for X-ray diffraction studies were obtained by ether diffusion into a solution of the title compound in DMF after several days at room temperature. Yield, 0.29 g (73%). (found: C, 54.16; H, 4.49; N,9.63. Calcd.: C, 54.22; H, 4.55; N, 9.58)

### Refinement

All H atoms were visible in difference Fourier maps and were subsequently refined in a riding-model approximation with C—H distances ranging from 0.93 to 0.97Å and *U*_iso_(H) = 1.2 *U*_eq_(C) or *U*_iso_(H) = 1.5*U*_eq_(C_methyl_).

### Crystal data

**Table:**

[CdBr_2_(C_30_H_26_N_4_S)]·C_3_H_7_NO	*F*(000) = 816
*M**_r_* = 819.92	*D*_x_ = 1.674 Mg m^−^^3^
Monoclinic, *P*2_1_/*m*	Mo *K*α radiation, λ = 0.71073 Å
Hall symbol: -P 2yb	Cell parameters from 3975 reflections
*a* = 9.7437 (8) Å	θ = 2.2–27.3°
*b* = 16.7792 (14) Å	µ = 3.23 mm^−^^1^
*c* = 10.5931 (9) Å	*T* = 296 K
β = 110.029 (1)°	Block, yellow
*V* = 1627.1 (2) Å^3^	0.36 × 0.32 × 0.28 mm
*Z* = 2	

### Data collection

**Table:**

Bruker APEXII area-detector diffractometer	3305 independent reflections
Radiation source: fine-focus sealed tube	2742 reflections with *I* > 2σ(*I*)
graphite	*R*_int_ = 0.027
ω scans	θ_max_ = 26.0°, θ_min_ = 2.1°
Absorption correction: multi-scan (*SADABS*; Bruker, 2006)	*h* = −11→12
*T*_min_ = 0.390, *T*_max_ = 0.465	*k* = −20→20
9062 measured reflections	*l* = −13→6

### Refinement

**Table:**

Refinement on *F*^2^	Secondary atom site location: difference Fourier map
Least-squares matrix: full	Hydrogen site location: inferred from neighbouring sites
*R*[*F*^2^ > 2σ(*F*^2^)] = 0.029	H-atom parameters constrained
*wR*(*F*^2^) = 0.077	*w* = 1/[σ^2^(*F*_o_^2^) + (0.0374*P*)^2^ + 0.7961*P*] where *P* = (*F*_o_^2^ + 2*F*_c_^2^)/3
*S* = 1.05	(Δ/σ)_max_ < 0.001
3305 reflections	Δρ_max_ = 0.78 e Å^−^^3^
211 parameters	Δρ_min_ = −0.64 e Å^−^^3^
0 restraints	Extinction correction: *SHELXL97* (Sheldrick, 2008), Fc^*^=kFc[1+0.001xFc^2^λ^3^/sin(2θ)]^-1/4^
Primary atom site location: structure-invariant direct methods	Extinction coefficient: 0.0025 (4)

### Special details

**Table:**

Geometry. All e.s.d.'s (except the e.s.d. in the dihedral angle between two l.s. planes) are estimated using the full covariance matrix. The cell e.s.d.'s are taken into account individually in the estimation of e.s.d.'s in distances, angles and torsion angles; correlations between e.s.d.'s in cell parameters are only used when they are defined by crystal symmetry. An approximate (isotropic) treatment of cell e.s.d.'s is used for estimating e.s.d.'s involving l.s. planes.
Refinement. Refinement of *F*^2^ against ALL reflections. The weighted *R*-factor *wR* and goodness of fit *S* are based on *F*^2^, conventional *R*-factors *R* are based on *F*, with *F* set to zero for negative *F*^2^. The threshold expression of *F*^2^ > σ(*F*^2^) is used only for calculating *R*-factors(gt) *etc*. and is not relevant to the choice of reflections for refinement. *R*-factors based on *F*^2^ are statistically about twice as large as those based on *F*, and *R*- factors based on ALL data will be even larger.

### Fractional atomic coordinates and isotropic or equivalent isotropic displacement parameters (Å^2^)

**Table:**

	*x*	*y*	*z*	*U*_iso_*/*U*_eq_	Occ. (<1)
Br1	0.53761 (5)	0.2500	0.42042 (5)	0.05529 (15)	
Br2	0.95622 (6)	0.2500	0.72708 (5)	0.05828 (15)	
C1	1.0172 (3)	0.16482 (17)	0.2726 (3)	0.0434 (7)	
H1A	0.9562	0.1832	0.1844	0.052*	
H1B	1.1010	0.1377	0.2630	0.052*	
C2	0.9329 (3)	0.10786 (16)	0.3253 (3)	0.0343 (6)	
C3	0.9688 (3)	−0.01196 (18)	0.1929 (3)	0.0410 (7)	
H3A	1.0153	−0.0620	0.2295	0.049*	
H3B	1.0400	0.0207	0.1714	0.049*	
C4	0.8399 (3)	−0.02804 (19)	0.0656 (3)	0.0444 (7)	
C5	0.7349 (5)	0.0278 (3)	0.0121 (4)	0.0949 (16)	
H5	0.7429	0.0777	0.0523	0.114*	
C6	0.6156 (6)	0.0109 (4)	−0.1025 (5)	0.117 (2)	
H6	0.5439	0.0493	−0.1376	0.140*	
C7	0.6033 (5)	−0.0607 (3)	−0.1629 (4)	0.0825 (13)	
H7	0.5234	−0.0716	−0.2395	0.099*	
C8	0.7068 (5)	−0.1163 (3)	−0.1119 (3)	0.0691 (11)	
H8	0.6985	−0.1656	−0.1539	0.083*	
C9	0.8260 (4)	−0.1008 (2)	0.0029 (3)	0.0545 (8)	
H9	0.8967	−0.1398	0.0376	0.065*	
C10	0.8378 (3)	−0.00630 (16)	0.3597 (3)	0.0343 (6)	
C11	0.7924 (3)	−0.08440 (17)	0.3631 (3)	0.0432 (7)	
H11	0.8186	−0.1248	0.3157	0.052*	
C12	0.7067 (3)	−0.09879 (19)	0.4403 (3)	0.0491 (7)	
H12	0.6721	−0.1501	0.4436	0.059*	
C13	0.6703 (3)	−0.03818 (19)	0.5139 (3)	0.0468 (7)	
H13	0.6136	−0.0506	0.5662	0.056*	
C14	0.7157 (3)	0.03915 (18)	0.5110 (3)	0.0421 (7)	
H14	0.6912	0.0791	0.5603	0.051*	
C15	0.8005 (3)	0.05523 (17)	0.4308 (3)	0.0351 (6)	
C16	0.6054 (6)	0.2500	1.0757 (6)	0.0683 (14)	
H16	0.5334	0.2500	1.1148	0.082*	
C17	0.4073 (8)	0.2500	0.8662 (7)	0.111 (3)	
H17A	0.3912	0.2763	0.7819	0.166*	0.50
H17B	0.3553	0.2776	0.9152	0.166*	0.50
H17C	0.3728	0.1961	0.8502	0.166*	0.50
C18	0.6637 (11)	0.2500	0.8761 (10)	0.133 (3)	
H18A	0.6547	0.2014	0.8260	0.199*	0.50
H18B	0.7607	0.2540	0.9405	0.199*	0.50
H18C	0.6452	0.2946	0.8158	0.199*	0.50
Cd1	0.81788 (3)	0.2500	0.46873 (3)	0.04091 (11)	
N1	0.8612 (2)	0.12589 (13)	0.4073 (2)	0.0359 (5)	
N2	0.9223 (2)	0.02893 (13)	0.2940 (2)	0.0355 (5)	
N3	0.5618 (5)	0.2500	0.9435 (5)	0.0682 (12)	
O1	0.7309 (5)	0.2500	1.1506 (5)	0.1167 (18)	
S1	1.07918 (11)	0.2500	0.38353 (10)	0.0414 (2)	

### Atomic displacement parameters (Å^2^)

**Table:**

	*U*^11^	*U*^22^	*U*^33^	*U*^12^	*U*^13^	*U*^23^
Br1	0.0422 (3)	0.0582 (3)	0.0688 (3)	0.000	0.0232 (2)	0.000
Br2	0.0716 (3)	0.0552 (3)	0.0480 (3)	0.000	0.0204 (2)	0.000
C1	0.0503 (17)	0.0394 (16)	0.0495 (16)	−0.0028 (13)	0.0285 (14)	−0.0058 (13)
C2	0.0313 (13)	0.0363 (15)	0.0343 (13)	0.0002 (11)	0.0099 (11)	−0.0025 (11)
C3	0.0445 (16)	0.0412 (16)	0.0406 (15)	0.0036 (13)	0.0189 (13)	−0.0089 (12)
C4	0.0488 (17)	0.0510 (18)	0.0343 (15)	0.0001 (14)	0.0156 (13)	−0.0017 (12)
C5	0.096 (3)	0.082 (3)	0.070 (3)	0.035 (3)	−0.018 (2)	−0.023 (2)
C6	0.099 (4)	0.139 (5)	0.071 (3)	0.049 (4)	−0.023 (3)	−0.013 (3)
C7	0.067 (3)	0.132 (4)	0.041 (2)	−0.013 (3)	0.0088 (18)	−0.010 (2)
C8	0.083 (3)	0.081 (3)	0.0469 (19)	−0.034 (2)	0.027 (2)	−0.0184 (19)
C9	0.066 (2)	0.055 (2)	0.0441 (17)	−0.0117 (16)	0.0214 (16)	−0.0056 (14)
C10	0.0282 (13)	0.0381 (14)	0.0342 (14)	0.0013 (11)	0.0075 (11)	0.0008 (11)
C11	0.0428 (16)	0.0383 (16)	0.0468 (16)	0.0023 (13)	0.0131 (13)	0.0017 (13)
C12	0.0426 (16)	0.0401 (17)	0.0612 (19)	−0.0004 (13)	0.0134 (15)	0.0135 (14)
C13	0.0357 (15)	0.0560 (19)	0.0510 (18)	0.0053 (14)	0.0179 (14)	0.0176 (14)
C14	0.0362 (14)	0.0488 (17)	0.0434 (16)	0.0070 (13)	0.0164 (13)	0.0046 (13)
C15	0.0268 (13)	0.0409 (15)	0.0353 (14)	0.0014 (11)	0.0077 (11)	0.0006 (11)
C16	0.049 (3)	0.071 (4)	0.077 (4)	0.000	0.011 (3)	0.000
C17	0.075 (5)	0.161 (8)	0.077 (4)	0.000	0.001 (4)	0.000
C18	0.134 (8)	0.157 (9)	0.136 (7)	0.000	0.083 (6)	0.000
Cd1	0.04412 (19)	0.03567 (18)	0.0520 (2)	0.000	0.02820 (15)	0.000
N1	0.0345 (12)	0.0377 (12)	0.0394 (12)	−0.0018 (10)	0.0175 (10)	−0.0041 (10)
N2	0.0356 (12)	0.0363 (12)	0.0355 (12)	−0.0006 (10)	0.0132 (10)	−0.0036 (9)
N3	0.057 (3)	0.073 (3)	0.073 (3)	0.000	0.020 (2)	0.000
O1	0.062 (3)	0.149 (5)	0.116 (4)	0.000	0.002 (3)	0.000
S1	0.0379 (5)	0.0342 (5)	0.0486 (6)	0.000	0.0104 (4)	0.000

### Geometric parameters (Å, °)

**Table:**

Br1—Cd1	2.6004 (6)	C10—C15	1.397 (4)
Br2—Cd1	2.6038 (6)	C11—C12	1.376 (4)
C1—C2	1.488 (4)	C11—H11	0.9300
C1—S1	1.817 (3)	C12—C13	1.399 (5)
C1—H1A	0.9700	C12—H12	0.9300
C1—H1B	0.9700	C13—C14	1.375 (4)
C2—N1	1.322 (3)	C13—H13	0.9300
C2—N2	1.360 (3)	C14—C15	1.399 (4)
C3—N2	1.468 (3)	C14—H14	0.9300
C3—C4	1.519 (4)	C15—N1	1.385 (3)
C3—H3A	0.9700	C16—O1	1.208 (7)
C3—H3B	0.9700	C16—N3	1.316 (7)
C4—C5	1.360 (5)	C16—H16	0.9300
C4—C9	1.374 (4)	C17—N3	1.446 (8)
C5—C6	1.393 (6)	C17—H17A	0.9600
C5—H5	0.9300	C17—H17B	0.9600
C6—C7	1.347 (7)	C17—H17C	0.9600
C6—H6	0.9300	C18—N3	1.408 (9)
C7—C8	1.344 (6)	C18—H18A	0.9600
C7—H7	0.9300	C18—H18B	0.9600
C8—C9	1.389 (5)	C18—H18C	0.9600
C8—H8	0.9300	Cd1—N1	2.264 (2)
C9—H9	0.9300	Cd1—N1^i^	2.264 (2)
C10—N2	1.380 (3)	Cd1—S1	2.9784 (11)
C10—C11	1.387 (4)	S1—C1^i^	1.817 (3)
			
C2—C1—S1	111.48 (19)	C12—C13—H13	119.1
C2—C1—H1A	109.3	C13—C14—C15	117.2 (3)
S1—C1—H1A	109.3	C13—C14—H14	121.4
C2—C1—H1B	109.3	C15—C14—H14	121.4
S1—C1—H1B	109.3	N1—C15—C10	109.2 (2)
H1A—C1—H1B	108.0	N1—C15—C14	130.7 (3)
N1—C2—N2	111.7 (2)	C10—C15—C14	120.1 (3)
N1—C2—C1	125.7 (2)	O1—C16—N3	125.8 (6)
N2—C2—C1	122.6 (2)	O1—C16—H16	117.1
N2—C3—C4	111.3 (2)	N3—C16—H16	117.1
N2—C3—H3A	109.4	N3—C17—H17A	109.5
C4—C3—H3A	109.4	N3—C17—H17B	109.5
N2—C3—H3B	109.4	H17A—C17—H17B	109.5
C4—C3—H3B	109.4	N3—C17—H17C	109.5
H3A—C3—H3B	108.0	H17A—C17—H17C	109.5
C5—C4—C9	118.4 (3)	H17B—C17—H17C	109.5
C5—C4—C3	121.5 (3)	N3—C18—H18A	109.5
C9—C4—C3	120.1 (3)	N3—C18—H18B	109.5
C4—C5—C6	120.4 (4)	H18A—C18—H18B	109.5
C4—C5—H5	119.8	N3—C18—H18C	109.5
C6—C5—H5	119.8	H18A—C18—H18C	109.5
C7—C6—C5	120.6 (5)	H18B—C18—H18C	109.5
C7—C6—H6	119.7	N1—Cd1—N1^i^	133.74 (11)
C5—C6—H6	119.7	N1—Cd1—Br1	103.31 (6)
C8—C7—C6	119.7 (4)	N1^i^—Cd1—Br1	103.31 (6)
C8—C7—H7	120.2	N1—Cd1—Br2	102.82 (6)
C6—C7—H7	120.2	N1^i^—Cd1—Br2	102.82 (6)
C7—C8—C9	120.6 (4)	Br1—Cd1—Br2	109.732 (19)
C7—C8—H8	119.7	N1—Cd1—S1	69.50 (5)
C9—C8—H8	119.7	N1^i^—Cd1—S1	69.50 (5)
C4—C9—C8	120.3 (4)	Br1—Cd1—S1	152.80 (3)
C4—C9—H9	119.9	Br2—Cd1—S1	97.46 (3)
C8—C9—H9	119.9	C2—N1—C15	106.0 (2)
N2—C10—C11	131.9 (3)	C2—N1—Cd1	126.29 (18)
N2—C10—C15	105.4 (2)	C15—N1—Cd1	127.02 (17)
C11—C10—C15	122.7 (3)	C2—N2—C10	107.6 (2)
C12—C11—C10	116.4 (3)	C2—N2—C3	128.0 (2)
C12—C11—H11	121.8	C10—N2—C3	123.7 (2)
C10—C11—H11	121.8	C16—N3—C18	120.8 (6)
C11—C12—C13	121.7 (3)	C16—N3—C17	119.8 (5)
C11—C12—H12	119.2	C18—N3—C17	119.4 (6)
C13—C12—H12	119.2	C1^i^—S1—C1	103.7 (2)
C14—C13—C12	121.9 (3)	C1^i^—S1—Cd1	93.83 (10)
C14—C13—H13	119.1	C1—S1—Cd1	93.83 (10)
			
S1—C1—C2—N1	25.0 (4)	N1^i^—Cd1—N1—C2	5.0 (3)
S1—C1—C2—N2	−154.3 (2)	Br1—Cd1—N1—C2	128.6 (2)
N2—C3—C4—C5	42.8 (5)	Br2—Cd1—N1—C2	−117.2 (2)
N2—C3—C4—C9	−136.2 (3)	S1—Cd1—N1—C2	−23.9 (2)
C9—C4—C5—C6	0.8 (7)	N1^i^—Cd1—N1—C15	−164.51 (14)
C3—C4—C5—C6	−178.2 (5)	Br1—Cd1—N1—C15	−40.9 (2)
C4—C5—C6—C7	−0.8 (9)	Br2—Cd1—N1—C15	73.3 (2)
C5—C6—C7—C8	0.2 (9)	S1—Cd1—N1—C15	166.6 (2)
C6—C7—C8—C9	0.4 (7)	N1—C2—N2—C10	0.2 (3)
C5—C4—C9—C8	−0.3 (5)	C1—C2—N2—C10	179.7 (2)
C3—C4—C9—C8	178.7 (3)	N1—C2—N2—C3	170.8 (2)
C7—C8—C9—C4	−0.3 (5)	C1—C2—N2—C3	−9.7 (4)
N2—C10—C11—C12	−179.7 (3)	C11—C10—N2—C2	179.8 (3)
C15—C10—C11—C12	0.4 (4)	C15—C10—N2—C2	−0.3 (3)
C10—C11—C12—C13	−1.4 (4)	C11—C10—N2—C3	8.7 (4)
C11—C12—C13—C14	1.2 (5)	C15—C10—N2—C3	−171.4 (2)
C12—C13—C14—C15	0.1 (4)	C4—C3—N2—C2	−100.7 (3)
N2—C10—C15—N1	0.3 (3)	C4—C3—N2—C10	68.5 (3)
C11—C10—C15—N1	−179.8 (2)	O1—C16—N3—C18	0.000 (6)
N2—C10—C15—C14	−179.0 (2)	O1—C16—N3—C17	180.000 (4)
C11—C10—C15—C14	0.9 (4)	C2—C1—S1—C1^i^	−128.05 (17)
C13—C14—C15—N1	179.8 (3)	C2—C1—S1—Cd1	−33.2 (2)
C13—C14—C15—C10	−1.1 (4)	N1—Cd1—S1—C1^i^	131.08 (12)
N2—C2—N1—C15	0.0 (3)	N1^i^—Cd1—S1—C1^i^	−27.04 (12)
C1—C2—N1—C15	−179.5 (3)	Br1—Cd1—S1—C1^i^	52.02 (10)
N2—C2—N1—Cd1	−171.33 (16)	Br2—Cd1—S1—C1^i^	−127.98 (10)
C1—C2—N1—Cd1	9.2 (4)	N1—Cd1—S1—C1	27.03 (12)
C10—C15—N1—C2	−0.2 (3)	N1^i^—Cd1—S1—C1	−131.08 (12)
C14—C15—N1—C2	179.0 (3)	Br1—Cd1—S1—C1	−52.02 (10)
C10—C15—N1—Cd1	171.06 (17)	Br2—Cd1—S1—C1	127.98 (10)
C14—C15—N1—Cd1	−9.8 (4)		

Symmetry codes: (i) *x*, −*y*+1/2, *z*.

### Hydrogen-bond geometry (Å, °)

**Table:**

*D*—H···*A*	*D*—H	H···*A*	*D*···*A*	*D*—H···*A*
C1—H1A···O1^ii^	0.97	2.38	3.004 (5)	122

Symmetry codes: (ii) *x*, *y*, *z*−1.
